# F-18 fluorodeoxyglucose positron emission tomography and/or computed tomography findings of an unusual breast lymphoma case and concurrent cervical cancer: a case report

**DOI:** 10.1186/1752-1947-4-282

**Published:** 2010-08-20

**Authors:** Nghi C Nguyen, Christopher N Hueser, Aarti Kaushik, Hussein R Farghaly, Medhat M Osman

**Affiliations:** 1 Department of Radiology, Division of Nuclear Medicine, Saint Louis University, 3635 Vista Avenue (at Grand Avenue), Saint Louis, MO, 63110, USA; 2 Department of Internal Medicine, Division of Hematology and Oncology, Saint Louis University, 3635 Vista Avenue (at Grand Avenue), Saint Louis, MO, USA

## Abstract

**Introduction:**

Breast lymphoma accounts for less than 1% of all non-Hodgkin's lymphomas and approximately 0.1% of all breast neoplasms. Most breast lymphomas are classified as diffuse large B-cell lymphomas or as mucosa associated lymphoid tissue lymphomas. Concurrent cases of breast lymphoma and cervical cancer are extremely rare.

**Case presentation:**

We report a case of a 46-year-old woman of unknown ethnic origin diagnosed with concurrent diffuse large B-cell lymphoma of the breast and squamous cell cancer of the cervix that was detected and followed with F-18 fluorodeoxyglucose (FDG) positron emission tomography and/or computed tomography (PET/CT). The metastatic pattern of this case of breast lymphoma is similar to that of a typical metastatic breast carcinoma. These findings have never been described in the literature. PET/CT also demonstrated an incidentally intense FDG focus in the uterine cervix ultimately leading to the pathologic diagnosis of squamous cell carcinoma of the uterine cervix. An appropriate staging of breast lymphoma and cervical cancer with FDG PET/CT is important because of therapeutic consequence. This case report and review of the literature highlights the role of FDG PET/CT in staging and restaging of both breast lymphoma and cervical cancer.

**Conclusions:**

We report a case of a breast lymphoma with a metastatic pattern similar to that of typical metastatic breast carcinoma. The FDG PET/CT scan also diagnosed a rare case of concurrent breast lymphoma and cervical cancer. This concurrence has not been reported previously in the medical literature.

## Introduction

Breast lymphoma accounts for less than 1% of all cases of patients with non-Hodgkin's lymphomas (NHL) [1] and approximately 0.1% of all cases of patients with breast neoplasms [2]. Most breast lymphomas are either classified as diffuse large B-cell (DLBC) lymphomas (as with the case of our patient) or as mucosa associated lymphoid tissue (MALT) lymphomas. The prevalence of breast lymphoma is much less compared to that of cervical cancer. In the USA, there were an estimated 11,070 new cases of invasive cervical cancer in 2008. As a result, 3870 cancer-related deaths are expected. This represents approximately 1% of cancer deaths in women [3]. 18-Fluorodeoxyglucose (FDG) positron emission tomography and/or computed tomography (PET/CT) has been shown to be useful in the diagnosis, staging and restaging of various cancers with accuracies ranging from 80% to 90% [4].

We describe the staging and restaging findings of FDG PET/CT scans in a patient with synchronous breast lymphoma and cervical cancer and highlight this rare clinical occurrence. To the best of our knowledge, concurrent breast lymphoma and cervical cancer have not been reported in the literature.

## Case presentation

**Figure 1:**
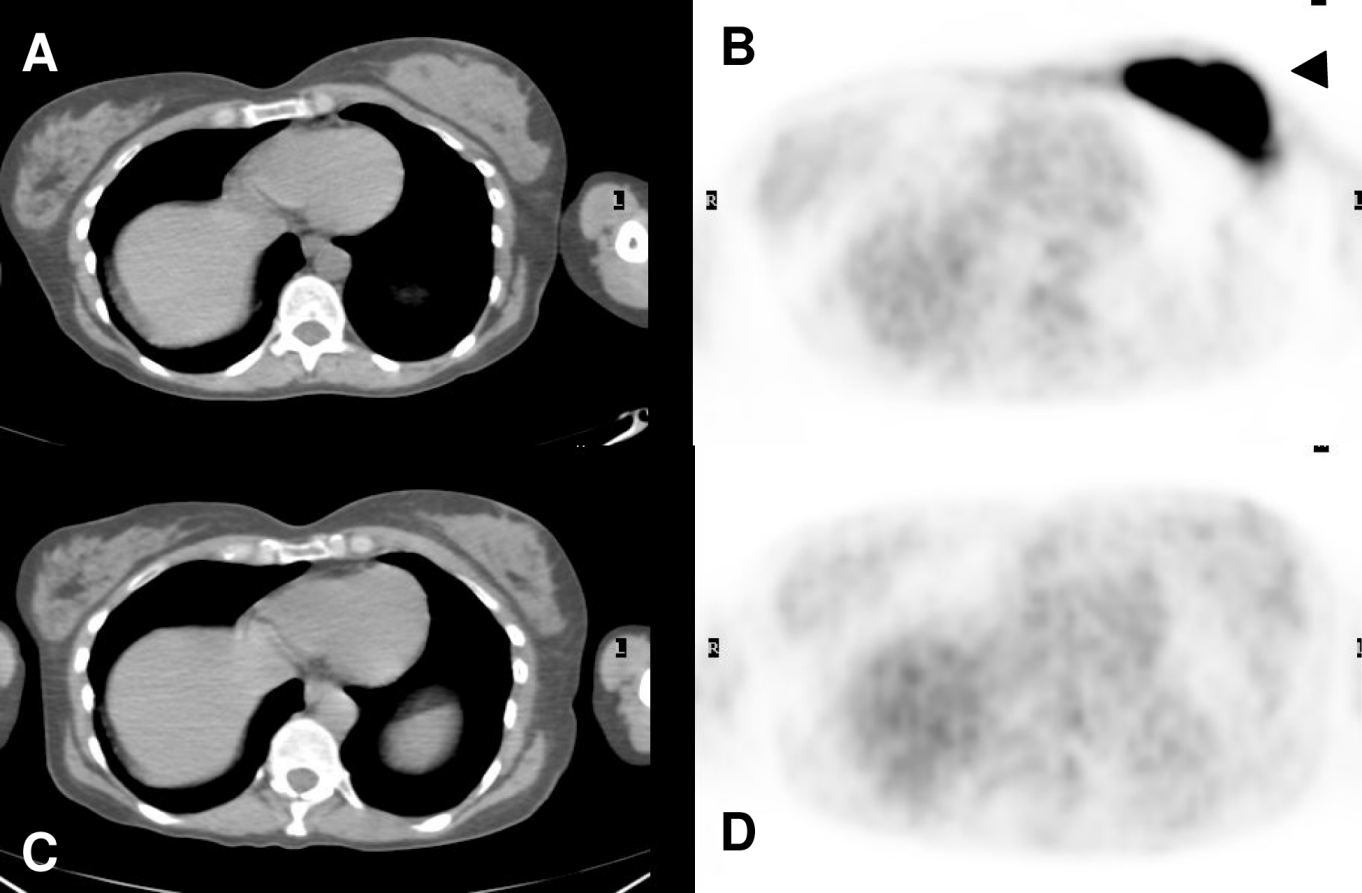
**(A) Axial computed tomography (CT) and (B) axial positron emission tomography (PET) images of pre-treatment PET/CT scan showed an intense FDG avid mass in the left breast (arrowhead)**. **(C) **Axial computed tomography (CT) and **(D) **axial positron emission tomography (PET) images of post-treatment PET/CT, three months after completion of chemotherapy, showed resolution of previously fludeoxyglucose (FDG) avid breast lesion.

**Figure 2:**
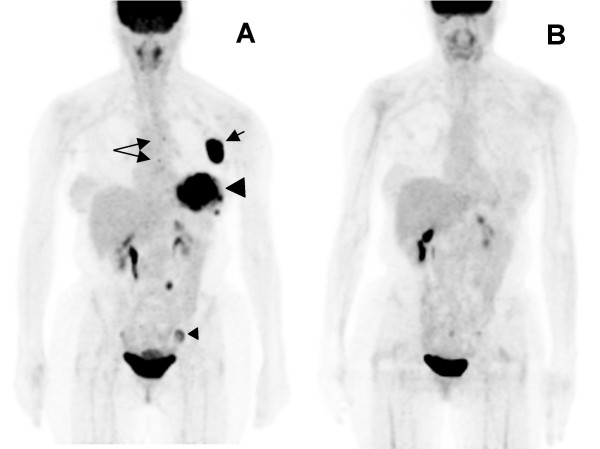
**Maximum intensity projection (MIP) PET images**: (A) Pre-therapy scan again showed the fludeoxyglucose (FDG) avid mass occupying almost the entire left breast (large arrowhead), further left axillary lymph node conglomerates (short arrow) and two left internal mammary lymph nodes (long arrows), all demonstrating high FDG avidity. An intense FDG focus in the right pelvic sidewall (small arrowhead) was a corpus luteal cyst verified by diagnostic computed tomography (CT) and ultrasound. Three months after completion of chemotherapy, a PET/CT scan **(B) **showed resolved FDG avidity of all previously described lesions in the left breast, left axilla and left internal mammary region, suggestive of complete remission of breast lymphoma. Spontaneous resolution of the corpus luteal cyst was also noticed.

**Figure 3:**
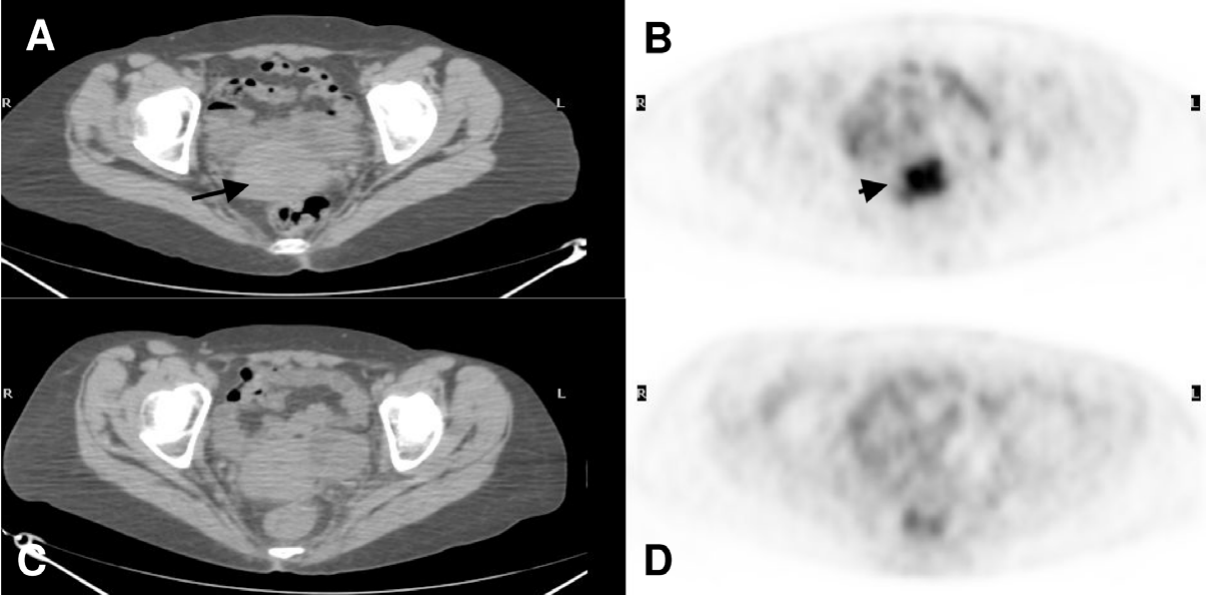
**(A) Axial computed tomography and (B) axial positron emission tomography images of pre-treatment PET/CT scan showed an intense FDG focus in the uterine cervix (short arrow) with soft tissue fullness on CT (long arrow)**. **(C) **Axial CT and **(D) **axial PET images of post-treatment PET/CT, three months after completion of chemotherapy, showed resolution of previously FDG avid cervical lesion.

A 46-year-old woman of unknown ethnic origin presented to her primary doctor with a one-month history of a painless left breast lump without associated nipple discharge that was noticed by the doctor on examination. She was otherwise healthy with no other relevant history. Physical examination revealed a large, non-tender, freely movable mass in the left breast and multiple enlarged lymph nodes in the left axilla. Our patient reported no systemic B symptoms such as fever or weight loss. A biopsy of her left breast mass revealed a DLBC lymphoma. Our patient was then referred for staging with F-18 FDG PET/CT that was acquired from base of skull to upper thigh with the CT being low-dose and unenhanced. The PET/CT scan revealed a 8 × 10 cm hyperdense and intensely FDG-avid mass occupying almost the entire left breast with maximum standard uptake value (SUV) of 21 (Figure 1). In addition, several left axillary lymph nodes measuring up to 5 cm in size and several left sub-centimeter internal mammary lymph nodes showed intense FDG avidity (Figure 2), with SUV values of 33 and 3.3. However, PET/CT findings were suggestive a breast carcinoma rather than a lymphoma, based on the location and distribution of the lesion. Because of the rarity of breast lymphoma, it would be unusual to consider metastatic breast lymphoma in the differential diagnosis of breast tumors. As a result, it would have been impossible to distinguish breast lymphoma from breast carcinoma through PET/CT. An incidental finding of intense FDG uptake in the uterine cervix, SUV of 8, led to the subsequent pathologic diagnosis of a previously unsuspected squamous cell carcinoma (Figure 3). The lesion appeared to involve the proximal third of the vagina and the corpus uterine, but a tumor extension to the parametrial soft tissue was not noticed.

Our patient underwent chemotherapy for NHL of the breast and cervical cancer as well as afterloading brachytherapy to the uterine cervix. A repeat FDG PET/CT three months after completion of therapy showed resolved FDG avidity of all previously described lesions (Figures 1, 2, 3).

## Discussion

### Breast lymphoma

DLBC lymphoma is an NHL that usually presents with a rapidly enlarging mass. Systemic B symptoms (that is fever and weight loss >10% of body weight) may occur in approximately 30% of patients [5]. Large B-cell lymphoma is the most common type of NHL; its prevalence is about 30% of all NHL patients. Also, large B-cell lymphoma accounts for approximately 40% of patients with extra-nodal NHL [2,6]. Extra-nodal sites may be of lung, pleura, thymus, breast, spleen, liver, pancreas, musculoskeletal system, or central nervous system [2]. Primary breast lymphoma is a rare disease and presented only 0.1% of the more than 25,000 primary malignant tumors of the breast treated during a 30-year period in a single institution [6].

There are only a few reports of FDG-PET findings of extra-nodal breast lymphoma [7-10]. Kumar *et al*. reported the findings of FDG-PET in a case of a patient with DLBC lymphoma that presented as intense and diffuse FDG uptake in dense breast tissue and was not detectable by diagnostic CT [7]. Bakheet *et al*. reported a patient with breast lymphoma mass that had intense FDG uptake in the rim and photopenic center suggestive a tumor with central necrosis [8]. Nihashi *et al*. described the FDG uptake as intense, round and homogeneous, but, unfortunately, there were no CT images for correlation [9]. Another case reported a patient with concurrent breast lymphoma and multiple nodular adenosis [10]. In that case, an FDG PET scan after one cycle of chemotherapy showed diffuse moderate FDG uptake in the right breast which might have reflected good response to therapy based on FDG intensity. In the current case, a large, intensely FDG avid, hyperdense tumor mass infiltrated almost the entire soft tissue of the left breast which was not described previously. In addition, the FDG avid lymph nodes in the ipsilateral axillary and internal mammary regions showed a metastatic pattern similar to that of typical metastatic breast cancer. These findings have not been reported in the literature.

The role of FDG PET in the diagnosis, staging and restaging of lymphoma has been established [2,11]. Integrated PET/CT increases the sensitivity and specificity compared to FDG PET alone. In Hodgkin's lymphoma or high-grade NHL, the sensitivity of PET/CT and contrast-enhanced CT for lymph node involvement was found to be 94% and 88%, respectively, while the specificity was 100% and 86%, respectively [11]. For extra-nodal disease, PET/CT and contrast-enhanced CT had a sensitivity of 88% and 50%, and a specificity of 100% and 90% [11]. The degree of FDG uptake can distinguish indolent from aggressive NHL [12]. An SUV >10 was found to have high likelihood for aggressive disease. In our patient, SUV was 21 in the breast mass and there was associated locoregional lymphadenopathy suggestive of aggressive disease. Early FDG PET/CT scan can be carried out after first-line chemotherapy to increase the prognostic value by assessing the degree of interval SUV decrease, with event-free survival improving from 65% to 76% in patients with DLBC lymphoma when quantitative SUV analysis was added to a visual assessment [13].

The Ann Arbor staging system developed in 1971 for Hodgkin's lymphoma was adapted for staging of NHL [14]. Based on the present FDG PET/CT findings, our patient had stage II disease because there was involvement of two lymph node regions on the same side of the diaphragm besides the primary breast lesion.

### Cervical cancer

The incidental detection of a second malignancy in cancer patients undergoing FDG PET/CT staging is not uncommon [15]. However, the FDG PET/CT scan diagnosed a rare case of concurrent breast lymphoma and cervical cancer that has never been reported in the literature. It seems unlikely, though, that the breast lymphoma and the cervical cancer of our patient are caused by one or the other.

The International Federation of Gynecologists and Obstetricians (FIGO) in collaboration with the World Health Organization (WHO) and the International Union Against Cancer (IUCC) are the most common staging systems for cervical cancer [16]. The FIGO staging system is largely based upon physical examination. Thus, a good pelvic examination is important. Tumor size and parametrial involvement are best assessed by rectovaginal examination. Colposcopy, cystoscopy, and proctoscopy can be used to assess adjacent areas. Optional procedures include ultrasound, CT, magnetic resonance imaging (MRI), and FDG PET or PET/CT, and can be of value for treatment planning.

FDG PET has been used in initial staging and monitoring of therapy in patients with cervical cancer [17]. In a review article, the sensitivity and specificity for pelvic involvement with newly diagnosed cervical cancer were 79% and 99% for FDG-PET, and 72% and 96% for MRI; for CT the sensitivity was 47%, the specificity could not be accurately determined. For para-aortic node metastasis, FDG-PET had a sensitivity of 84%, and a specificity of 95% [17]. The diagnostic accuracy of FDG PET in lymph node staging, however, might be lower in women with early stage diseases [18]. This is partly attributed to the low-dose and unenhanced CT of the PET/CT scan that is sub-optimal for detecting sub-centimeter nodal disease. Of note, the CT was sub-optimal in the current patient as well. But PET/CT scan is increasingly being carried out with intravenous contrast media.

In our patient, FDG PET/CT findings suggested a T2 tumor that involved the proximal third of the vagina and the corpus uterine. There was no evidence of parametrial tumor invasion. An intensely FDG avid soft tissue density seen in the left pelvis was thought to be either lymph node metastasis or physiologic ovarian FDG uptake. Subsequent ultrasound and diagnostic contrast-enhanced CT confirmed the presence of a corpus luteal cyst that sometimes may cause false-positive interpretation because of the FDG avidity [19]. Based on imaging findings, the cervical cancer was T2N0M0, stage II. The degree of FDG uptake has prognostic significance in cervical cancer and was found to negatively correlate with treatment response and prognosis [20].

## Conclusions

This case report shows a breast lymphoma case of a patient with a metastatic pattern similar to that of typical metastatic breast carcinoma. Also, the FDG PET/CT scan diagnosed an extremely rare case of concurrent breast lymphoma and cervical cancer. FDG PET/CT has advantage over other imaging modalities because of its whole-body scanning that offers detection of metastasis and any previously unknown malignancy.

## Consent

Written consent for publication could not be obtained despite all reasonable attempts. All efforts have been made to protect the identity of the patient and there is no reason to believe the patient would object to publication.

## Competing interests

The authors declare that they have no competing interests.

## Authors' contributions

NCN is the senior author and was involved in collecting patient information, reviewing the literature and doing the final proofreading of the manuscript. CNH was involved in discussion and editing of the manuscript. HRF and AK helped capture and prepare the images. MMO contributed to the discussion, editing and proofreading of the manuscript. All authors read and approved the final manuscript.
